# The Gulf War Women’s Health Cohort: Study Design and Protocol

**DOI:** 10.3390/ijerph17072423

**Published:** 2020-04-02

**Authors:** Benjamin E. Ansa, Kimberly Sullivan, Maxine H. Krengel, Vahé Heboyan, Candy Wilson, Stacey Iobst, Steven S. Coughlin

**Affiliations:** 1 Institute of Public and Preventive Health, Augusta University, Augusta, GA 30912, USA; 2 Applied Health Sciences Program, Augusta University, Augusta, GA 30912, USA; 3 Department of Environmental Health, Boston University School of Public Health, Boston, MA 02118, USA; tty@bu.edu; 4 Research Service, VA Boston Healthcare System, Boston, MA 02130, USA; Maxine.Krengel@va.gov; 5 Department of Interdisciplinary Health Sciences, College of Allied Health Sciences, Augusta University, Augusta, GA 30912, USA; vheboyan@augusta.edu; 6 Uniformed Services University Graduate School of Nursing, Bethesda, MD 20814, USA; phdcandy@gmail.com; 7 Henry M. Jackson Foundation at the Uniformed Services University Graduate School of Nursing, Bethesda, MD 20814, USA; stacey.iobst.ctr@usuhs.edu; 8 Department of Population Health Sciences, Medical College of Georgia, Augusta University, Augusta, GA 30912, USA

**Keywords:** Gulf War, Gulf War illness, chronic multisymptom illness, veterans, women

## Abstract

Military service and deployment affect women differently than men, underscoring the need for studies of the health of women veterans and their receipt of health care services. Despite the large numbers of women who served during the 1990–1991 Gulf War, few studies have evaluated Gulf War illness (GWI) and other medical conditions specifically as they affect women veterans of the 1991 Gulf War. The objectives of the Gulf War Women’s Health Cohort study are: (1) to establish the Gulf War women’s cohort (GWWC), a large sample of women veterans who served in the 1990–1991 Gulf War and a comparison group of women who served in other locations during that period; and (2) to provide current, comprehensive data on the health status of women who served during the 1990–1991 Gulf War, and identify any specific conditions that affect Gulf War women veterans at excess rates. The study will utilize both existing datasets and newly collected data to examine the prevalence and patterns of Gulf War Illness symptoms, diagnosed medical conditions, reproductive health, birth outcomes and other health issues among women who served during the Gulf War. The Gulf War Women’s Health Cohort study will address the need for information about the comprehensive health of women veterans who were deployed to the Gulf War, and other wars during the Gulf War era.

## 1. Introduction

Increased prevalence of reported health conditions and symptoms in musculoskeletal, neurological, pulmonary, gastrointestinal, and dermatological systems have been acknowledged among deployed veterans of the 1991 Gulf War (GW) era, when compared with veterans who did not deploy to the war [1,2,3,4,5]. These health conditions and symptoms, commonly known as Gulf War illness (GWI), are referred to by the Department of Veterans Affairs as “chronic multisymptom illness” [6].

Among the nearly 700,000 military personnel who served in the 1991 Gulf War, almost 7% (49,000) were women [7,8]. Military service and deployment affect women differently than men, underscoring the need for studies of the health of women veterans and their receipt of health care services across their lifespan [7,9]. Although in nearly three decades since the war, some studies have investigated the rates of GWI in female vs. male GW veterans, with results suggesting that GWI is more common in women GW veterans than their male counterparts [10,11], few studies have evaluated GWI and other medical conditions specifically as they affect women veterans of the 1991 Gulf War.

A small number of studies suggested excess rates of women’s health problems, e.g., breast cysts, abnormal pap smears, yeast infections, and bladder infections [7]. Several studies have identified significantly elevated rates of birth defects and adverse reproductive outcomes among GW veterans. Increased risks of ectopic pregnancies and spontaneous abortions were observed in some studies [12]. Overall, however, findings have varied with different study designs and sample sizes, with some studies showing elevated risks of stillbirths, miscarriages, and/or birth defects [13,14,15,16,17,18]. There remains a need to evaluate birth outcomes specifically among women GW veterans, in appropriate subgroups, e.g., by time-period of birth, by parental exposures and by other deployment characteristics.

Studies that have investigated the mental health of veterans revealed that the proportions of females with major depressive disorder, post-traumatic stress disorder, and anxiety disorder were higher among Gulf War veterans (35%, 23.7% and 17.1% respectively), compared to Gulf War era veterans (27.1%, 12.3% and 17.0% respectively) [9,19].

Theories about the pathobiology of GWI focus on neurological, immune, and endocrine mechanisms, which may be differentially altered in women by life course events from pregnancy to menopause. Menopause has effects on a number of organ systems including the cardiovascular, skeletal, nervous, and genitourinary systems. In addition, pregnancy complications, including pre-eclampsia and gestational diabetes can result in later life health effects, including cardiovascular disease and adult onset diabetes in at-risk women. Given that many female veterans who were deployed to the Gulf region are now middle-aged, they may be at risk for these later lifespan health outcomes. These outcomes have been poorly studied to date and thus more research on this topic is needed.

Based on the reasons enumerated above, there remains a paramount need to evaluate the health status across the life course of women GW veterans in appropriate subgroups, e.g., by hazardous and risky exposures, and by other deployment characteristics. The objectives of this study are: (1) to establish the Gulf War women’s cohort (GWWC), a large sample of women veterans who served in the 1990-1991 Gulf War and a comparison group of women who served in other locations during that time period; and (2) to provide current, comprehensive data on the health status of women who served during the 1990-1991 Gulf War, and identify any specific conditions that affect GW women veterans at excess rates.

The specific aims include: (1) to assemble the GWWC using data collected from 955 to 1420 women GW veterans and an additional 680 to 854 women veterans who were not deployed to the Persian Gulf, who participated in previous and ongoing population studies of 1990–1991 Gulf War era veterans. (2) To conduct a multimodal health survey to provide data on the current health status of the subset of previously surveyed GW era women veterans who are eligible to be re-contacted (with a projected 200 completed surveys) and comparisons between GW deployed veterans and non-deployed GW era veterans. (3) To provide comprehensive data on veteran-reported pregnancy and birth outcomes among GW and GW era women veterans. (4) To evaluate GWI and other high interest health outcomes in women veteran subgroups, including subgroups identified by (a) veterans’ deployment characteristics (e.g., locations and exposures, branch of service), and (b) veterans’ age and menopausal status (e.g., pre-, peri-, and post-menopause subgroups). (5) Where possible, to provide a longitudinal assessment of changes in GW era women veterans’ health over time, using baseline data collected in the original population studies from which the current cohort sample is drawn. (6) To examine sex differences in GWI including female to male differences in the frequency of symptoms associated with GWI and the overall prevalence of GWI among GW female and male veterans.

## 2. Materials and Methods

### 2.1. Study Design

The study involves the reanalysis of existing data from epidemiologic studies of the health of Gulf War veterans and a survey of women included in the Ft. Devens Cohort Study.

### 2.2. Study Sites

The lead site is Augusta University. VA Boston Healthcare and the Boston University School of Public Health Data Coordinating Center will be collecting survey data. All required Institutional Review Board (IRB) approvals will be obtained at each site before project implementation. The study team holds monthly conference telephone calls, and all non-compliance and/or unanticipated problems associated with the study protocol or applicable requirements will be reported in accordance with local policy.

### 2.3. Study Participants and Inclusion Criteria

All of the study participants served during the 1991 Gulf War and participated in previously conducted epidemiologic studies of the health of Gulf War veterans. Participants will be located using their last known mailing address, with contact information updates using information from multiple sources, including the U.S. Postal Service and commercial locating services. The VA Boston Healthcare System is responsible for contacting women veterans who previously participated in the Ft. Devens Cohort (FDC) Study. The Ft. Devens, MA Cohort of GW veterans is one of the few longitudinal cohorts of GW veterans and is the longest running cohort of GW veterans [18]. The most recent Time 5 resurvey began in 2013 and 448 participants who had data for at least one medical condition responded to the FDC Reunion Survey (47 women).

### 2.4. Procedure

The study will utilize both existing datasets (Specific Aim 1) and newly collected data (Specific Aim 2) to examine the prevalence and patterns of GWI symptoms, diagnosed medical conditions, reproductive health, birth outcomes (Specific Aim 3), and other health issues across the lifespan, including hypertension, cardiovascular risk and diabetes among women who served during the Gulf War. The project will provide a comprehensive picture of the health of women GW veterans. This includes assessment of current health status, changes in health symptoms and conditions over time, and possible differences in health outcomes associated with specific experiences and exposures during the war (Specific Aim 4). It will allow for an assessment of GWI symptom patterns that may be specific to women veterans, a determination of diagnosed medical conditions among women veterans, and an evaluation of changes in women’s health over time, including changes potentially associated with menopause and early reproductive complications and subsequent risk for increased age-related health outcomes (Specific Aim 5). The proposed study will utilize data from multiple studies in order to establish the GWWC. In studies that have retained personal identifiers and for which it is feasible to obtain IRB permission to re-contact subjects, current data will be collected on women’s current health symptoms, GWI (defined by both Kansas and CDC CMI criteria) [20], diagnosed medical conditions, and reproductive outcomes and complications (e.g., still births, ectopic pregnancies, birth defects, pre-eclampsia, gestational diabetes). For all studies, re-analyses of existing data will be conducted to focus on health outcomes specifically affecting women. This will allow for the frequency and nature of GWI symptoms to be better characterized among women veterans. Female to male differences in GWI will be examined in some of the studies, in order to determine whether GWI manifests differently in women (Specific Aim 6).

In the study, a re-survey of a longitudinal cohort will be included (Ft. Devens cohort (FDC) Reunion Survey). Dr. Maxine Krengel at the VA Boston Healthcare System and Boston University School of Medicine, and Dr. Kimberly Sullivan at the Boston University School of Public Health, recently conducted a follow-up survey of male and female Army veterans who were deployed to the Persian Gulf and participated in the Ft. Devens cohort Study. Through the use of longitudinal data, where health symptoms were first measured one year after deployment and then repeatedly over the past 19 years, they are examining symptom trajectories, i.e., patterns of change over time. The use of longitudinal data allows consideration of relapsing, remitting, and late-emerging symptoms, in order to refine case definitions of GWI and for a clearer understanding of the potential causes for these symptom trajectories (i.e., genetic predisposition, environmental exposures, prior treatment efficacy). Several questions related to women’s health have been asked in surveys of the cohort, including difficulty conceiving, number of births, whether a child was born with a birth defect, stillbirths, uterine or ovarian tumors, hysterectomy, menopause, amenorrhea, vaginal yeast infections, premenstrual symptoms, pain during intercourse, difficulty achieving orgasm, Depo-Provera shots, and breast disease. The most recent survey of this cohort ended by September 2016.

In 1991, a total of 1290 subjects from the Ft. Devens cohort participated in a postal survey, in which the prevalence of 52 symptoms and risk factors for reported symptoms were examined. The survey questionnaire included questions about background and demographic variables, GW duty service (active, reserve/National Guard, military rank), health outcomes (health functioning, general symptoms, medical conditions, and symptoms associated with multiple chemical sensitivity), numerous environmental exposures associated with GW deployment, and alcohol use. About 60% of the respondents met the CDC criteria for chronic multisymptom illness (CMI). Female gender, lower levels of education, self-reported use of a medical clinic in the Gulf, ingestion of anti-nerve gas pills, anthrax vaccination, tent heaters, and exposure to oil fire smoke, and chemical odors were related to CMI in logistic regression analyses [7]. Women from this cohort will be resurveyed as part of the GWWC. The Ft. Devens cohort was one of the first studies to report a 1.8 times higher risk of GWI in women veterans [9].

Dr. Beth Unger at the Centers for Disease Control and Prevention (CDC) in Atlanta provided archived records from the CDC Air Force Study, which included women. The study data, which contain no personal identifiers or biological specimens, have been made available to Augusta University for use in the proposed collaborative study. In November 1994, the VA, DoD, and the Pennsylvania Department of Health requested that CDC investigate a report of unexplained illnesses among members of an Air National Guard unit who were GW veterans. After an initial investigation of 59 GW veterans, which involved standardized interviews and physical examinations, a larger sample of GW veterans (*n* = 3927) were surveyed in 1995, who were members of the index unit and three comparison units in Pennsylvania and Florida. After excluding 204 who were younger than 17 years during the GW, 1163 (31.2%) were GW veterans and 2560 (68.8%) had not been deployed. In addition to general health history, the respondents were asked about the frequency, duration, and severity of 35 symptoms and possible exposures during deployment. Birth outcomes were not asked about. In all units, the prevalence of each of 13 chronic symptoms was significantly greater (*p* < 0.05) among persons deployed to the GW than among those not deployed.

In addition to the CDC Air Force study and extending data collection among participants in the Ft. Devens Cohort, we are proposing to include data from VA’s Cooperative Studies Program (CSP) 585 Gulf War Biorepository and Health Survey pilot study (which ended in May 2016) [1,21]). Veterans were eligible to participate in the VA’s Cooperative Studies Program (CSP) 585 Gulf War Biorepository and Health Survey pilot study, if they had served in the U.S. Uniformed Services during the period from August 1990–July 1991, regardless of deployment or combat status. The stratified recruitment panel was obtained from the Department of Defense Manpower Data Center (DMDC) and was drawn from a total population of 4,966,117 veterans who served during August 1990–July 1991. A subset of only the 301 women CSP 585 veterans (291 DMDC and 10 self-nominated) was included for the current proposed study. A data usage agreement was finalized with the principal investigator of the CSP 585 pilot study, Dr. Dawn Provenzale at the VAMC in Durham, NC, and Dr. Coughlin at Augusta University.

Dr. Sullivan is currently the PI and leader of a DoD funded Gulf War Illness Consortium (GWIC) that brings together diverse experts from 9 institutions to study the pathobiology and treatment development for GWI. GWIC clinical studies include data collection from three study sites in Boston, Central Texas and Miami. Clinical study co-investigators include Drs. Nancy Klimas, Lea Steele and Maxine Krengel. The GWIC cohort will include study surveys of 300 GW veterans (about 20% women) that will be shared for use in the currently proposed study.

Taken overall, the minimum sample size expected for this study is 6,835 male and female veterans. The number of women who will be included in GWWC is anticipated to be 560 to 800 women GW veterans and an additional 680 to 959 women veterans who were not deployed to the Persian Gulf. This includes 1290 Army GW veterans from the Ft. Devens Cohort, 300 GW veterans from the Boston GWI Consortium (20% women), over 1318 GW veterans from CSP 585 pilot study (23.7% women), 3927 GW veterans from the CDC USAF study (which included about 522 women).

New data collection for the GWWC will involve telephone interviews and postal surveys of women (with a projected 200 completed surveys), including questions on women’s health previously utilized in surveys conducted by the investigators at VA, VA Boston Healthcare system, and Boston University. De-identified data will be compiled and analyzed by Augusta University investigators in collaboration with the other study sites.

### 2.5. Survey Questionnaire Design

A survey questionnaire was developed based upon questionnaires used in previous surveys (Ft. Devens Cohort and Kansas study). Questions are included about a variety of topics including marital status, employment status, educational attainment, general health status, symptoms, medical conditions diagnosed or treated by a physician, use of health care services, and reproductive outcomes and complications. The respondents will be asked about the frequency, duration, and severity of a comprehensive list of about 40 symptoms, partly to ensure that both the CDC and Kansas criteria for GWI can be used in the analysis. They will also be asked if they had ever been diagnosed or treated by a physician for any of about 25 medical and psychiatric conditions, and when each reported condition had developed. An open-ended question will also be included about additional medical or psychiatric conditions they may have had diagnosed or been treated for. Data will be collected on gender-specific symptoms and medical conditions that occur in women and about adverse reproductive outcomes and complications (spontaneous abortions, stillbirths, ectopic pregnancies, pre-term births, birth defects, pre-eclampsia and gestational diabetes).

For women veterans, questions will be included about the use of hormone replacement therapy, whether they ever had an abnormal Pap smear (and when), whether they have had a hysterectomy, whether they were pregnant or nursing in the past year, and whether they had menstrual periods in the past year. An open-ended question will also be included about additional gynecological problems they may have had.

The survey will take about 30 to 45 min to complete and will be carefully pilot tested in a small sample of veterans before use.

### 2.6. Interviewing Process

All subjects will be assigned a participant ID number. Following institutional review board (IRB) approval, the veterans will be sent an advance letter signed by the PI that describes the study, requests their participation, and lets them know that, if they agree to participate, they will have the option of being interviewed by telephone or by filling out and returning a postal survey questionnaire. The letter will include a copy of the IRB-approved consent form. A postcard will be included to allow them to opt out of the survey.

New data collection for the GWWC will involve postal surveys and telephone surveys (CATI telephone survey questionnaires) of women from the Ft. Devens Study (with a projected 200 completed surveys). Web-based data collection will be undertaken if a high response rate is not achieved using telephone and postal surveys. The questions and content of the online survey questionnaire would be identical to the postal survey.

#### 2.6.1. Computer-assisted Telephone Interviews

Veterans who do not opt out will be contacted by telephone, in order to invite them to complete the survey and obtain their informed consent. Computer-assisted telephone interviews will be used for this purpose. Attempts will be made to reach the veterans, calling at different times on different days of the week. Once a potential respondent is reached by telephone and their identity is verified by the interviewer, the consent form will be read to the veteran and, if they agree to be interviewed, the interviewer will proceed with the survey questions using predetermined prompts and procedures. Telephone interview responses will be captured by a REDCap electronic questionnaire.

#### 2.6.2. Postal Surveys

For respondents who have not opted out or who cannot be reached by telephone or for whom a telephone number is unavailable, data will be collected using postal survey questionnaires developed in a TELEForm optical character recognition system. All subjects will have an assigned participant ID number. A sequential mailing protocol will be followed using a modified Dillman method. A letter, signed by the principal investigator, will be sent to each veteran that requests their participation and includes two copies of a consent form, a postal survey questionnaire, and a pre-addressed, stamped return envelope. Three weeks after the survey mailing, a postcard thank you/reminder card will be sent. After 6 weeks, veterans who have not completed and returned the questionnaire or refused consent will be sent a second mailing with a cover letter, consent form, questionnaire, and return envelope, followed by a postcard thank you/reminder card. After 12 weeks, veterans who have not completed and returned the questionnaire or refused consent will be sent a second mailing with a cover letter, consent form, questionnaire, and return envelope, followed by a postcard thank you/reminder card. Paper questionnaires will include a scannable barcode identifying the veteran and whether it was from the first, second, or third wave of the survey.

Survey responses will be checked for completeness and then coded and scanned using Teleform software for entry into an electronic database. All electronic data will be stored on a secure server with access controlled by unique login names and passwords; paper forms will be stored in a locked cabinet in the principal investigator’s locked office. Personal identifiers needed for tracking purposes will be stored separately from the research data, whether electronic or paper. All investigators and staff who have access to patient information will be required to complete required human subjects research ethics training. The research team will conduct annual evaluations for quality assurance and data integrity purposes.

#### 2.6.3. Web-Based Survey

If a high response rate is not achieved using telephone and postal surveys, web-based surveys will be undertaken, using methods previously used by the Boston University School of Public Health Data Coordinating Center to survey veterans in the Ft Devens Reunion Survey [18]. In collecting information from the respondents, personally identifying information is kept separate from health information and the records are only linked with the subjects’ unique study identification number. Veterans who have not participated in the Gulf War Women Health Cohort Study telephone or postal survey, and who have not refused consent, will be sent a letter inviting them to complete the online survey. They will be provided with a unique web personal identification number and the website URL. The online survey will begin by asking them to read the consent form. The questions that are included in the online survey (if the survey is undertaken) will be identical to those in the postal survey questionnaire.

### 2.7. Quality Assurance

All survey data collected as part of the proposed study will be carefully monitored for completeness. If a veteran returns two copies of the questionnaire, the most complete questionnaire will be selected for inclusion. A scannable bar code will identify the veteran and whether the questionnaire was from the first, second, or third wave. The quality of the data will be maximized through pre-coded responses and computerized internal consistency checks and range checks of specified values. The use of CATI interviews and barcodes and Teleform software for scanning paper questionnaires will maximize the accuracy of computer databases. Any information from paper questionnaires that requires manual entry into computer databases (e.g., information from open-ended responses) will be entered twice, in order to check for data entry errors and to resolve any discrepancies. Personally identifying information, such as names and addresses, will be kept separate from survey responses and will be kept under lock and key.

### 2.8. Type of Data Collected

Information will be collected about self-reported symptoms, medical conditions, and military deployment-related exposures.

### 2.9. Outcomes Measures

The main outcomes of interest include GWI defined using the CDC and Kansas criteria. Other outcomes of interest include self-reported health status (excellent, very good, good, fair, or poor), functional status, individual symptoms and medical conditions, including those that are gender-specific and only occur among women. Table 1 provides a list of self-reported medical conditions diagnosed or treated by a doctor that will be examined in the proposed study, including their date of onset (before 1990 or after 1990).

Other outcomes of interest that will be examined in the analysis include spontaneous abortions, stillbirths, ectopic pregnancies, pre-term births, and birth defects. A large number of individual symptoms will be examined in the proposed study, including their severity (mild, moderate, or severe) and date of onset (before 1990 or after 1990), and whether they have been a problem in the past six months or past year.

### 2.10. Risk Factors, Potential Confounding Variables, and Effect Modifiers

Gender is a key risk factor in this study. Gender may also modify the effect of other risk factors such as exposures during military deployment. Several variables will be examined as risk factors, potential confounding variables or effect modifiers including age, marital status, educational attainment, military rank (officer, enlisted), branch of service, and component (active, reserves, National Guard). In analyses of data for women, additional variables that will be examined in the analysis include use of hormone replacement therapy and menopausal status.

### 2.11. Statistical Analysis

Cross-tabulations will be used to analyze data on the frequency and patterns of veteran reported chronic symptoms and medical conditions diagnosed by healthcare providers (Specific Aim 2a). Chi-square and Fisher’s exact tests will be performed to examine the statistical significance of observed associations. Prevalence odds ratios will be obtained with 95% confidence intervals. Additional analyses will be stratified on branch of service, component, or study cohort. Logistic regression will be used to obtain the prevalence odds ratios associated with chronic symptoms and medical conditions. These outcomes will be assessed in relation to military, deployment, and demographic characteristics using stratified analyses and included in multivariable models as appropriate. Military characteristics (GW deployment status, rank) and demographic variables (age category, sex, education) will be included in the models. Analyses of medical conditions and symptoms will be repeated while focusing on conditions that began after 1990.

The prevalence of GWI defined by the CDC and Kansas criteria (Specific Aim 2b) will be examined by obtaining proportions of veterans who have combinations of symptoms that meet the criteria, along with 95% confidence intervals. This will be done separately for GW and GW era veterans. Prevalence odds ratios will then be obtained in which GWI is the dichotomous dependent variable and the covariates include age categories, sex, deployment status, military rank, and study cohort. Two or more design variables will be used for nominal variables that have three or more categories (for example, branch of service, component). In determining whether the Kansas criteria for GWI are met, cases will be excluded if the veterans had one or more medical conditions (cancer other than non-melanoma skin cancer, diabetes, heart disease other than high blood pressure, chronic infectious disease, liver disease, lupus, multiple sclerosis, stroke, bipolar disorder, schizophrenia) or had been hospitalized since the Gulf War for depression, post-traumatic stress disorder, or alcohol or drug dependence.

The prevalence of female-specific health symptoms and medical conditions (Specific Aim 2c) will be examined by obtaining proportions of women veterans who have combinations of symptoms that meet the criteria, along with 95% confidence intervals. This will be done separately for GW and GW era veterans. Additional analyses will be stratified on branch of service, component, or study cohort. Prevalence odds ratios will then be obtained, in which GWI is the dichotomous dependent variable; and covariates include age categories, deployment status, branch of service, component, military rank, and study cohort.

Analyses of general health and functional status, use of healthcare services, and hospitalizations (Specific Aim 2d) will consist of descriptive analyses (frequency distributions and cross-tabulations) of the data performed using SAS. Additional analyses will be stratified by deployment status and sex. Both chi-square and Fisher’s exact tests will be used to examine the statistical significance of observed associations. In order to adjust for age and study cohort, the Mantel–Haenzel procedure will be used.

Analyses of veteran-reported pregnancy and birth outcomes among GW and GW era women veterans (Specific Aim 3) will also consist of frequency distributions and cross-tabulations of the data, stratified by deployment status. Additional analyses will be stratified on branch of service, component, or study cohort. Further analyses will be stratified by whether or not the veteran met criteria for GWI using the CDC and Kansas case definitions. The Mantel–Haenzel procedure and logistic regression methods will be used to adjust for age, study cohort, and other potential confounding variables.

The prevalence of GWI in women veteran subgroups (Specific Aim 4) will be examined by obtaining proportions of veterans who have combinations of symptoms that meet the CDC and Kansas criteria of GWI, along with 95% confidence intervals. This will be done separately for GW and GW era veterans. Stratified analyses will be completed in order to examine subgroups of women defined by deployment characteristics (location, exposures, branch of service). Similar analyses will be completed while stratifying on age, menopausal status, (pre-, peri-, and post-menopause subgroups), and study cohort. The Mantel–Haenzel procedure and logistic regression methods will be used to adjust for study cohort and other potential confounding variables.

Changes in GW and GW era women veterans’ health over time (Specific Aim 5) will be examined using baseline and follow-up data obtained from women veterans in the Ft. Devens cohort and Kansas study. These analyses will be limited to women veterans who participated in both the baseline and follow-up surveys. Cross-tabulations of the data will initially be performed. Chi-square and Fisher’s exact tests will be used to examine the statistical significance of observed temporal associations. Prevalence odds ratios associated with chronic symptoms, GWI, and other chronic medical conditions will be obtained using logistic regression. Military characteristics (deployment status) and demographic variables (age at baseline, education) will be included in the models as appropriate.

To examine sex differences in the frequency of symptoms associated with GWI and in the prevalence of GWI and other chronic medical conditions among female and male veterans (Specific Aim 6), cross-tabulations of the data will be completed, together with chi-square and Fisher’s exact tests, to examine the statistical significance of observed associations. Additional analyses will be stratified on GW deployment status, sex, branch of service, component, rank, or study cohort. Prevalence odds ratios associated with chronic symptoms and medical conditions will be obtained using logistic regression. Effect modification by sex will initially be examined in exploratory cross-tabulations of the data and then by including interaction terms in the models. Log-likelihood ratio tests will be used to determine levels of statistical significance. Military characteristics (GW deployment status, rank) and demographic variables (age category, sex, education) will be included in the models. Analyses of medical conditions and symptoms will focus on conditions that began after 1990.

After female veterans in the Ft. Devens Study have been resurveyed, it will be possible to conduct longitudinal analyses from two time points to determine whether the scores on a variable increased or decreased over time. The changes in scores (Y_2-1_) will be calculated using the longitudinal modeling with logistic regression. One score will be subtracted from another, where the score at the first time point (Y_1_) is subtracted from the score at the second time point (Y_2_): Y_2-1_ = Y_2_ − Y_1_.

Pooled analyses of data from two or more study cohorts will be completed to examine data collected at about the same time period, and the outcome and explanatory variables are measured in a similar fashion. When pooled analyses are undertaken, results will first be examined for each study cohort separately so that the heterogeneity of study results can be assessed. Survey data collected from veterans included in the Ft. Devens Cohort will allow for pooled analyses, because the procedures for collecting new data via postal questionnaire or telephone interview are identical.

#### Power Analysis

Sample size calculations were based upon an array of assumptions, taking into account likely response rates and attrition. Based upon literature review, the proportion of GW women who have GWI were estimated to be 35–40%, taking into account branch of service and differences across studies. The percentage of women who report a history of other medical conditions of interest will likely vary widely between 5–50%, depending upon the current prevalence of common chronic conditions, such as hypertension, overweight, and obesity. Based upon a type I error rate of 5% and statistical power of 0.80, a sample of 800 GW women veterans and an equal number of non-deployed women veterans would allow for the detection of a 20% difference in the proportion of women who have CMI, high blood pressure, or overweight/obesity (Specific Aim 1). Based upon a type I error rate of 5%, and power of 0.80, a sample of 800 GW female veterans and a substantially larger sample of male GW veterans will allow for the detection of a 10% difference in the proportions of women and men who have GWI (Specific Aim 6).

Additional sample size calculations were carried out for a two-tailed test on proportions (P1, P2). For a two-tailed test on proportions, with a type I error rate of 5%, power of 0.80, and where P1 = 0.15 and P2 = 0.30, 134 women per group (or 268 women total) would be needed to detect this difference (Specific Aims 2–5). For ordinal variables, the statistical power to detect meaningful differences across groups will be greater.

## 3. Ethical Considerations

This study protocol has been reviewed and approved by the Augusta University IRB and the IRBs at other participating institutions. There are no known risks to participants in this survey, other than potential, minor psychological distress. The research team will be cognizant of participants’ response/reactions to questions and medical diagnoses and will provide more detailed information and/or support if and when necessary. Potential risks due to breaches of confidentiality will be minimized by strict adherence to measures for protecting the confidentiality of the data.

Personally identifying information will be kept separate from survey responses under lock and key, and only members of the study team will have access to the data. Study data and PDF files of scanned survey questionnaires (without personal identifiers) will be transmitted to the Boston University School of Public Health Data Coordinating Center (BU DCC) using the BU DCC’s secure STP transfer site. Personal identifiers will not be included in data files or PDF files sent to the BU DCC using the secure server. A BU DCC secure transfer site will be used to transfer the overall study data set (analytic file) from BU to Augusta University and the other participating institutions for statistical analysis.

Personally identifying information from the Ft. Devens Study will only be available to Dr. Maxine Krengel at the VA Boston Healthcare System and her research assistant, and will not be shared with other study investigators. Personally identifying information from the CSP 585 pilot study is not being provided to GWWC Study investigators, only de-identified data. The other existing datasets that are being assembled to create the GWWC consist of de-identified data.

The potential benefits of the study include increased knowledge of the prevalence of GWI and symptoms among GW female veterans, factors associated with symptoms, and female/male differences in GWI. There is no direct benefit to participants in this study.

## 4. Conclusions

The Gulf War Women’s Health Cohort Study will address the need for information about the comprehensive health of women veterans that were deployed to the Gulf War, and other wars during the Gulf War era. The study is likely to contribute importantly to the understanding of key women veterans’ health concerns related to deployment during the Gulf War. A broad range of women’s health issues will be addressed, including adverse reproductive outcomes and complications (spontaneous abortions, stillbirths, ectopic pregnancies, pre-term births, birth defects, pre-eclampsia and gestational diabetes).

The study will provide important information about changes in physical and mental health as women veterans advance in age. A particular goal of the study is to establish a cohort of women veterans who can be invited to participate in IRB approved clinical research studies. Potential topics for these studies include symptom-based conditions, such as Gulf War illness and chronic fatigue syndrome; chronic pain, headache, traumatic brain injury, and other neurological conditions; and chronic conditions that are prevalent in both veteran and non-veteran populations as people reach middle age or older age (for example, diabetes, cardiovascular disease, rheumatoid arthritis, chronic pulmonary conditions).

Results from some studies related to this project have been published. A recent study of the rates of chronic medical conditions in 1991 Gulf War veterans using the Ft. Devens cohort revealed that female GW veterans had lower odds of developing high blood pressure and higher odds of developing diabetes when compared with age-matched females from the 2013–2014 National Health and Nutrition Examination Survey (NHANES) [22]. An analysis of the CDC Air Force Study revealed that the prevalence of mild-to-moderate and severe cases of CMI was 39% and 6%, respectively, among GW veterans, compared with 14% and 0.7% among 2520 non-deployed veterans. Although no physical examination, laboratory, or serologic findings identified cases, veterans who met the case definition had significantly diminished functioning and well-being [23]. Results to date from the re-analysis of the Cooperative Studies Program 585 Gulf War Era Cohort and Biorepository indicate that GW women showed significantly increased symptom reporting in 7 of the 34 symptoms queried, compared with non-deployed GW-era women veterans ([24], in press). The GW deployed women were also significantly more likely to report more total symptoms than the GW-era women, with more than half of the GW women reporting more than 21 total current health symptoms. Younger and non-white GW women veterans were particularly likely to report more total health symptoms.

## Figures and Tables

**Table 1:** Medical conditions that will be examined in the Gulf War Women’s Cohort study.

Asthma Allergies High blood pressure High cholesterol Migraine headaches Seizures Arthritis (and what type) Any skin condition (and what type) Chronic bronchitis Lung disease (and what type) Heart disease (and what type) Diabetes Thyroid problem (and what type) Gestational diabetes Pre-eclampsia Cerebrovascular disease Peripheral vascular disease	Stomach or intestinal problem (and what type) Skin cancer Any other cancer (and what type) Depression Alcohol or drug dependence Fibromyalgia Bladder infections Chronic fatigue syndrome Post-traumatic stress disorder Fertility problems Hysterectomy (women only) Menopausal status (women only) Breast lumps or cysts (women only) Yeast infections (women only) Abnormal Pap smear (women only) Lupus
